# Public knowledge of and attitudes toward genetics and genetic testing in Brunei Darussalam

**DOI:** 10.3389/fgene.2023.1181240

**Published:** 2023-04-24

**Authors:** Hazreana Jaya, Siti Nur Idayu Matusin, Aklimah Mustapa, Muhammad Syafiq Abdullah, Mas Rina Wati Haji Abdul Hamid

**Affiliations:** ^1^ Raja Isteri Pengiran Anak Saleha Hospital, Bandar SeriBegawan, Brunei; ^2^ Halalan Thayyiban Research Centre, Universiti Islam Sultan Sharif Ali, Tutong, Brunei; ^3^ Pengiran Anak Puteri Rashidah Sa’adatul Bolkiah (PAPRSB) Institute of Health Sciences, Universiti Brunei Darussalam, Gadong, Brunei

**Keywords:** genetics, genetic testing, public, knowledge, attitudes, perception

## Abstract

The world has been experiencing encouraging research in genetics, but current public knowledge, awareness, and perception of this area remain unknown for Brunei Darussalam. This study aimed to investigate the Brunei population’s genetics and genetic testing literacy, and their attitude toward them. A cross-sectional study was carried out targeting public population in Brunei Darussalam. Questionnaires on knowledge and attitudes were randomly distributed in frequented venues in the Brunei–Muara district and uploaded online for distribution through social media. Responses were scored and analyzed using appropriate statistical methods. Overall, the sample population (*n* = 474) comprised 75.7% female, 64.3% aged 18–29 years old, 39.7% with a bachelor’s degree, and 2.3% and 5.3% with a personal history and family history of genetic disease(s), respectively. Younger participants scored higher for disease-related questions and showed more concern on the impact of testing on employment but were more fearful of testing. Higher educational qualifications were associated with a higher knowledge score, a more optimistic view on DNA research, and less reluctance to take a genetic test for an untreatable disease. Participants with a personal history of genetic disease(s) were more knowledgeable and displayed higher curiosity. Participants with a family history of genetic disease(s) were also more knowledgeable and would want testing even for an untreatable disease. Significantly less was known about the social consequences of testing compared to the medical possibilities. Investigating the knowledge and attitudes of the population is vital preceding efforts toward national adaptation of genetic testing, keeping in mind the various obstacles and issues surrounding the subject.

## 1 Introduction

The world of genetics is constantly engaged in the discovery of the latest genes, propelling endless promises upon application of this information. Accompanied by the rising advancements in technology and analysis (Goodwin et al., 2016), research on genetics leads to critical understanding and efforts such as ascertaining risk factors (Haga et al., 2013) and imposing prevention and treatment of the diseases they cause. The expanding possibilities of this field have led to the growing interest around genetic testing. An example can be seen in hereditary breast cancer whereby the demand for genetic testing to predict hereditary risks is increasing since the discovery of *BRCA1* and *BRCA2* genes as breast cancer susceptibility genes, which account for 5%–10% of hereditary breast cancer cases (Miki et al., 1994; Wooster et al., 1994).

Knowledge on predisposition means the benefits of testing does not limit itself to the individual but ripples to members of the family. However, the excessive exposure on genetic testing has been influenced by the media, funding agencies, and scientific publication, creating unnecessary pressures to the scientific community (Caulfield and Condit, 2012) to prove its significance. Furthermore, it affects the patients or consumers (Haga et al., 2013), limiting their experience of its benefits and further translation of such testing. Thus, by exploring into individual’s knowledge and personal attitude toward genetic testing, it can be optimally exploited (Hietala et al., 1995; Cappelli et al., 1999). Such knowledge and attitude depend on the health literacy, which still contains gaps in interpreting genetic and genomic information (Lea et al., 2011). Nevertheless, public attitudes on genetics are commonly positive, depending on age, gender, and the educational level (Henneman et al., 2013; Vermeulen et al., 2013). However, some are ambivalent about family history’s risk assessment to prevent diseases (Vermeulen et al., 2013). Notably, acceptance of the genetic test’s result is a part of utilization, but understanding and correct interpretation of the results is also important (Haga et al., 2013).

Cancer is the leading cause of mortality in Brunei Darussalam, a small country located in Southeast Asia, with 21.4% risk of developing cancer before the age of 75 years, which is the second highest among countries in the region but considerably lower than Europe with 28.2% risk (Control U for IC, 2020). Cancer is a multifactorial and heterogeneous disease, involving genetics in its development. A study focusing on the contribution of genetics, specifically germline *BRCA1, BRCA2, TP53,* and *PALB2* mutations in breast cancer patients in Brunei Darussalam, showed that 4.2% of the patients were attributed to germline *BRCA2* mutations (Matusin, 2020). The study also highlighted the need for increasing public awareness on the contribution of genetics in breast cancer, as evidenced by the lack in knowledge of genetic testing and the effect of genetic variation in cancer development among patients and their family members (Matusin, 2020). Furthermore, there has not been any study on public knowledge of and attitudes toward genetics and genetic testing in Brunei Darussalam. Notably, any formal education on genetics in the country begins in high school, briefly touching on the nature of chromosome inheritance, the role of genes, and types of mutations.

This research aimed to investigate the Brunei population’s genetics and genetic testing literacy, and their attitude toward them. In this study, genetic literacy is defined as an individual, society, or nation’s knowledge level, or understanding of genetics and genomics (Boerwinkel et al., 2017). This includes knowledge about the structure, function, and behavior of genes; their inheritance patterns; and the way genetic information can be utilized to improve health (Boerwinkel et al., 2017). Moreover, genetic literacy also encompasses ethical, legal, and social implications of advances in genetics research and technology (Boerwinkel et al., 2017).Identifying the stance of the population of genetic testing, appropriate efforts can then be strategized for further actions such as education, counseling, and implementation, which ultimately aims to improve management of disease, patients, and patients’ family.

## 2 Materials and methods

### 2.1 Study design and population

This is a cross-sectional study conducted between September and December 2016, using convenient sampling, targeting public population residing in one out of four of the districts in Brunei Darussalam, the Brunei–Muara district. In accordance with the Department of Economic Planning and Statistics Brunei, the total Brunei population residing in the Brunei–Muara district in 2016 was 289,630 (JPKE D of EP and Brunei, 2018). Using a Raosoft sample size calculator (Raosoft, Inc. 2004; http://www.raosoft.com/samplesize.html) (Raosoft, 2021), assuming the expected prevalence of 5%, and providing a confidence level of 95%, with a margin of error of 5%, this study requires at least 384 responses to be collected. Questionnaires were randomly distributed to the public at frequented areas, including shopping complexes and multi-purpose stadiums, and collected immediately after responses were completed by participants. To ensure the target population size was reached, the questionnaire was also uploaded online for distribution via social media. The study population’s criteria included those with a minimum of 18 years of age and living in Brunei–Muara district.

### 2.2 Ethical approval

The study was approved by the PAPRSB Institute of Health Sciences (IHS) Research and Ethics Committee (IHSREC) of Universiti Brunei Darussalam (UBD). Before the survey, the informed written consent was obtained from each participant and they were informed that the research was voluntary, confidential, and anonymous as none of their personal details except for age was recorded.

### 2.3 Questionnaire design

**FIGURE 1:**
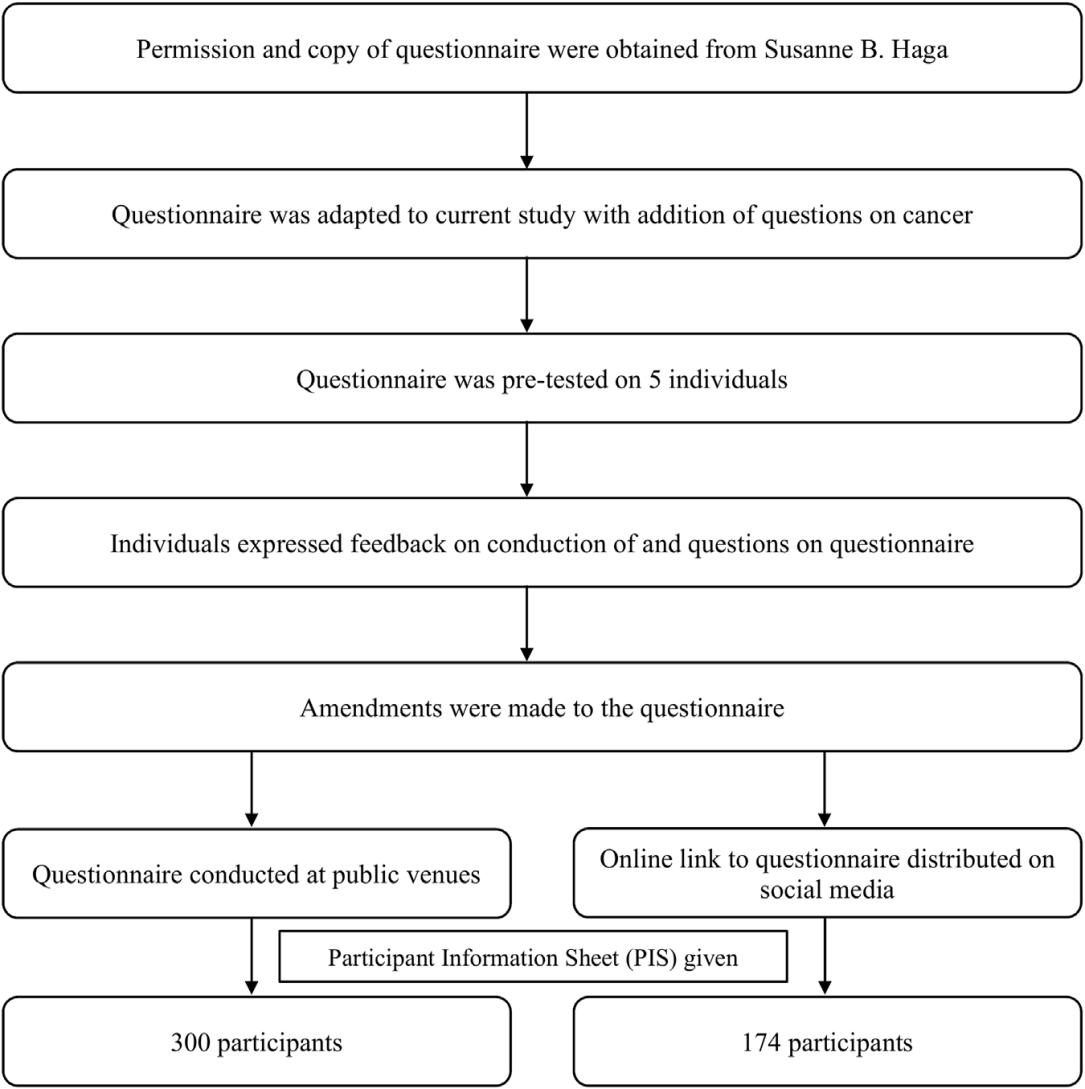
Flow chart of study materials and methods.

The questionnaire, obtained with permission from Haga et al., 2013, was slightly modified to include family and medical histories and change of certain words and phrases to be better understood by the Bruneian public. It was piloted with five individuals, followed by further amendments with simpler phrases and clearer instructions. The flow chart of the questionnaire and study designs is shown in Figure 1.

The adapted questionnaire included four parts with a total of 49 closed-ended questions. Part 1 (seven questions) was added to compile the participant’s sociodemographic data. Part 2 highlighted on the participant’s actual knowledge of genetics with 16 statements on genes, chromosomes, cells, and diseases in the dichotomous form, i.e., true/false. Part 3 (13 questions) focused on the perceived knowledge of genetics with nine and four statements on medical possibilities of genetic testing and social consequences of genetic testing, respectively, in trichotomous form, i.e., nothing/a little/sufficient. Notably, four additional questions were added in this section specifically exploring the participant’s knowledge on breast and colorectal cancers, and the genes responsible for them. The participant’s attitudes were assessed in Part 4 focused on 13 questions on prospects of DNA research and impacts of testing on self, family, and work. Part 4 was assessed using a five-point Likert scale ranging from 1 to 5, where 1 represented “totally disagree” and 5 represented “totally agree.”

Prior to starting the survey, the questionnaire was pre-tested on five respondents, with comments received to reconstruct and categorize some questions, and rephrase scientific terminologies. Responses received in the pre-test were not included in the final statistical analyses. The questionnaire was prepared in two languages, English and Malay, to ease public understanding.

### 2.4 Statistical analysis

A scoring system was used to analyze the questionnaire data. Questions in Part 2 of the questionnaire were marked based on correctness, 1 = correct and 0 = incorrect. The total score was then calculated and classified according to the following knowledge-level categories: inadequate = 0%–53%, moderate = 54%–66%, and adequate = 67%–100% (Haga et al., 2013). Questions in Part 3 were scored on a scale of 1 = none, 2 = a little, and 3 = a lot. Meanwhile, questions in Part 4 were graded on a five-point scale of 1 = totally disagree, 2 = disagree, 3 = neutral/do not know, 4 = agree, and 5 = totally agree.

The collected data were analyzed using Statistical Package for Social Sciences (SPSS) version 22.0 software. Descriptive statistics were used to summarize participants’ sociodemographic details, and percentage responses and mean scores for the remaining sections. Pearson chi-squared, Fisher’s exact, Independent t-, Kruskal–Wallis, one-way ANOVA *post hoc* (Scheffe), and Mann–Whitney tests were used to find associations between actual and perceived knowledge of genetics and attitudes with gender, ethnicity, age group, educational level, and genetic personal and family history. A *p*-value < 0.05 was considered statistically significant.

## 3 Results

### 3.1 Characteristics of participants

In total, 474 individuals were included in this study with a median age of 23 years (range 18-69). Notably, 75.7% of the participants were female, 84.2% identified as Malays, 64.3% aged 18 to 29 years old, and 39.7% had the educational level of a bachelor’s degree (Table 1). Eleven participants reported a personal medical history of genetic disease(s), while 25 participants had a family member(s) with a medical history of genetic disease(s). One out of 11 and one out of 25 participants who reported a personal and family history of genetic disease(s), respectively, stated that each of them have genetic diseases.

**TABLE 1:** Characteristics of participants (*n* = 474).

	*n* (%)		*n* (%)
**Gender**		**Medical history of other diseases** **^j^**	
Male	114 (24.1)	None	367 (77.4)
Female	359 (75.7)	Cardio and vascular diseases^d^	42 (8.9)
Missing response	1 (0.2)	Skin diseases^e^	9 (1.9)
**Ethnicity**		Gastrointestinal diseases^f^	5 (1.1)
Malay	399 (84.2)	Respiratory diseases^g^	17 (3.6)
Chinese	45 (9.5)	Other diseases^h^	7 (1.5)
Other	15 (3.2)	Missing response	27 (5.7)
Missing response	15 (3.2)	**Family history of genetic disease** **^j^**	
**Age (in years)**		None	449 (94.7)
18–29	305 (64.3)	Cancer^b^	15 (3.2)
30–39	66 (13.9)	Thalassemia	10 (2.1)
40–49	38 (8.0)	Other^c^	1 (0.2)
≥50	45 (9.5)	**Family history of other diseases** **^j^**	
Missing response	20 (4.2)	None	305 (64.3)
**Education**		Cardio and vascular diseases^d^	89 (18.8)
High school	79 (16.7)	Skin diseases^e^	7 (1.5)
College	71 (15.0)	Gastrointestinal diseases^f^	8 (1.7)
Bachelor’s degree	188 (39.7)	Respiratory diseases^g^	21 (4.4)
Postgraduate (master’s)	50 (10.5)	Neurologic diseases^i^	3 (0.6)
Others^a^	84 (17.7)	Other diseases^h^	2 (0.4)
Missing response	2 (0.4)	Missing response	43 (9.1)
**Medical history of genetic disease** **^j^**			
None	463 (97.7)		
Cancer^b^	3 (0.6)		
Thalassemia	8 (1.7)		
Other^c^	1 (0.2)		

^a^ Other education: Certificate, diploma, postgraduate (PhD), or not specified.

^b^ Cancer: breast, cervix, colorectal, endocrine, and prostate.

^c^ Other genetic diseases: color -blindness.

^d^ Cardiovascular diseases: anemia, atrial septal defect, diabetes mellitus, hyperlipidemia, hypertension, myocardial infarction, stroke, and other heart problems.

^e^ Skin diseases: eczema, psoriasis, and other skin diseases.

^f^ Gastrointestinal diseases: gall bladder cyst, gastric, hepatitis B, neonatal jaundice, renal failure, and ulcer.

^g^ Respiratory diseases: asthma and tuberculosis.

^h^ Other diseases: allergies, flu, glaucoma, gout, and PCOS (polycystic ovary syndrome)

^i^ Neurologic diseases: autism, degenerative motor neuron, muscle dystrophy, and Parkinson’s disease.

^j^ Category did not sum up to 100% due to ≥1 individual with ≥1 diseases.

### 3.2 Actual knowledge of genetics

The median score for all questions pertaining to actual knowledge of genetics (16 items) was 11 (range 0–16). The overall mean percentage of participants answering questions correctly was 69.4% (SD = 15.31), with a higher subsection percentage for questions on disease-related concepts (74.5%, SD = 15.80) compared to questions on scientific facts (67.9%, SD = 15.38), but no significant differences were noted between the two (*p* = 0.437) (Table 2).

**TABLE 2:** Actual knowledge of genetics.

Statement	Participants answering questions correctly (%)
Scientific facts (correct answer)	
1. A gene can be seen with a naked eye (false)	80.1
2. A gene is a disease (false)	74.8
3. A gene is a molecule that controls hereditary characteristics (true)	80.8
4. Genes are inside cells (true)	88.8
5. A gene is a piece of DNA (true)	78.7
6. A gene is a cell (false)	44.8
7. A gene is a part of a chromosome (true)	76.1
8. Different body parts include different genes (false)	44.2
9. Genes are bigger than chromosomes (false)	65.3
10. The genotype can be changed by humans (true)	53.7
11. It has been estimated that a person has 22,000 genes (true)	59.1
Subsection mean (SD)	67.9 (15.38) ^a^
Disease-related concepts (correct answer)	
12. Healthy parents can have a child with a hereditary disease (true)	76.6
13. Certain diseases are due to genes, environment, and lifestyle (true)	91.1
14. The carrier of a disease gene may be completely healthy (true)	75.6
15. All serious diseases are hereditary (false)	80.9
16. The child of a carrier of a disease gene is always also a carrier of the same disease gene (false)	48.5
Subsection mean (SD)	74.5 (15.80) ^a^
Overall mean (SD)	69.4 (15.31)

SD, standard deviation.

^a^ Differences between scientific facts and disease-related scores were not significant (*p* = 0.437).

Participants with a personal medical history of genetic disease attained a significantly higher score for all questions than participants without a personal history (*p* = 0.013), and participants with a family medical history of genetic disease scored significantly higher than participants without a family history (*p* = 0.012) (Table 3). The mean score for questions on disease-related concepts was significantly different among age groups (*p* = 0.002, Table 3). The *post hoc* test (Scheffe’s procedure) revealed that participants within 18-29 years old scored higher than participants aged 50 or above with mean scores of 3.6 (SD = 1.38) and 2.9 (SD = 1.65), respectively (Table 3). Significant differences in scores for disease-related concepts were also observed among the varying educational backgrounds of participants (*p* = 0.004, Table 3). Participants with a higher education level (bachelor’s degree or master’s degree) attained a greater score than participants with a lower education level (high school). No significant difference was noted in overall mean scores with respect to gender (*p* = 0.437) and ethnicity (*p* = 0.258) (Table 3).

**TABLE 3:** Statistical analysis for the respective variables pertaining to actual knowledge of genetics.

Variable	*n*	Actual knowledge					
		*Scientific facts*		*Disease-related*		*Overall*	
		Mean (SD)	*p*-value	Mean (SD)	*p*-value	Mean (SD)	*p*-value
**Gender**							
Male	114	6.7 (2.67)	0.779^a^	3.2 (1.54)	0.138^a^	10.0 (3.85)	0.437^a^
Female	359	6.8 (2.64)		3.5 (1.42)		10.3 (3.67)	
**Ethnicity**							
Malay	399	7 (3)	0.395^b^	4 (1)	0.663^b^	11 (4)	0.258^b^
Chinese	45	8 (3)		4 (2)		11 (4)	
Other	15	8 (2)		4 (2)		11 (2)	
**Age (in years)**							
18–29	305	7.0 (2.63)	0.111^c^	3.6 (1.38)	0.002^c,d^	10.6 (3.65)	0.018^c^
30–39	66	6.4 (2.39)		3.4 (1.47)		9.8 (3.40)	
40–49	38	6.5 (2.71)		3.0 (1.39)		9.4 (3.79)	
≥50	45	6.3 (2.79)		2.9 (1.65)		9.2 (3.97)	
**Education**							
High school	79	6.4 (2.15)	0.361^c^	2.9 (1.22)	0.004^c,e^	9.4 (2.67)	0.070^c^
College	71	6.8 (2.61)		3.6 (1.41)		10.4 (3.68)	
Bachelor’s degree	188	6.9 (3.06)		3.5 (1.60)		10.4 (4.43)	
Postgraduate (master’s)	50	7.4 (2.41)		3.8 (1.30)		11.2 (3.42)	
Others	84	6.6 (2.15)		3.3 (1.24)		9.9 (2.76)	
**Medical history of genetic disease**							
No	463	7 (3)	0.013^f^	4 (1)	0.404^f^	11 (4)	0.013^f^
Yes	11	9 (2)		4 (0)		13 (2)	
**Family history of genetic disease**							
No	449	7 (3)	0.017^f^	4 (1)	0.152^f^	11 (4)	0.012^f^
Yes	25	8 (4)		4 (1)		12 (4)	

SD, standard deviation.

^a^ Independent *t*-test.

^b^ Kruskal–Wallis test, values are presented as median (interquartile range).

^c^ One-way ANOVA test.

^d^ Age group 18–29 and ≥50 years old pair of mean score is significantly different by the *post hoc* test (Scheffe’s procedure).

^e^ Bachelor’s degree or Postgraduate (Master’s) and high school pairs of mean score are significantly different by the *post hoc* test (Scheffe’s procedure).

^f^ Mann–Whitney test, values are presented as median (interquartile range).

**FIGURE 2:**
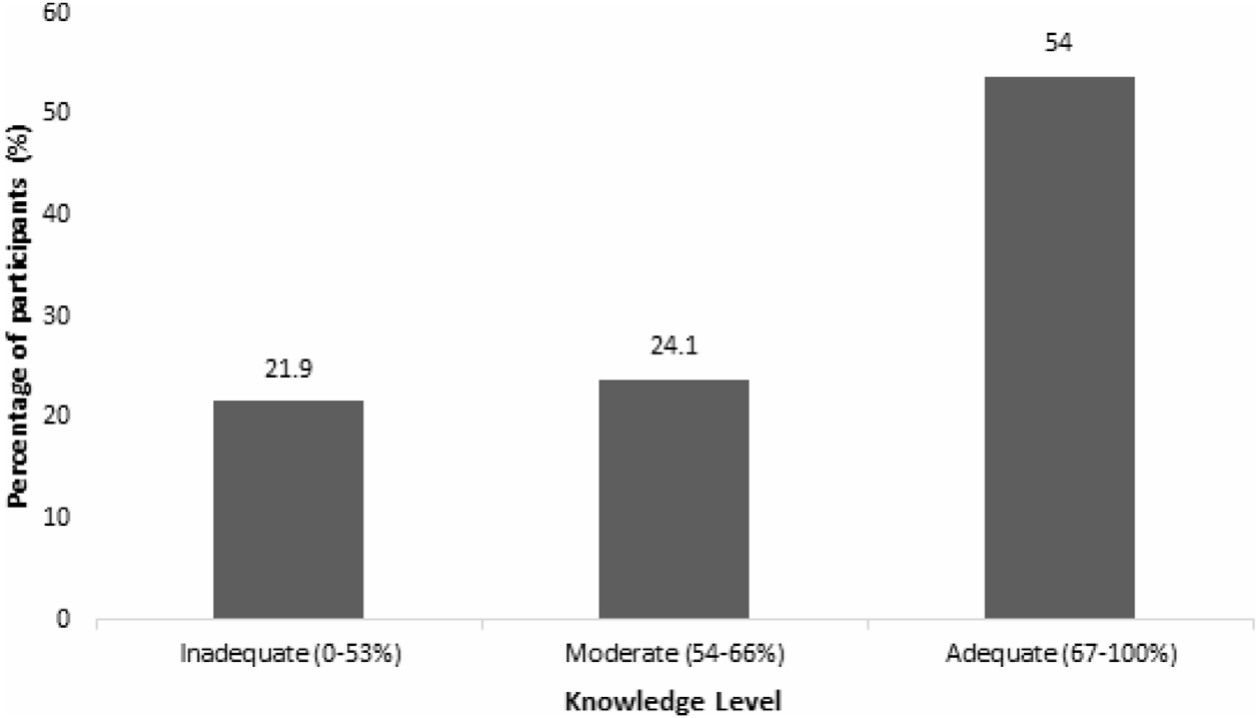
Distribution of the knowledge levels of the participants.

Majority of the participants (*n* = 256, 54.0%) have adequate knowledge of genetics (Figure 2). The actual knowledge of genetics among the participants can be strongly associated with their educational level (*p* = <0.001) and having personal history of genetic diseases (*p* = 0.038) (Table 4).

**TABLE 4:** Association between respective variables and participant’s knowledge level of genetics.

Variable	*n*	Knowledge level			*p*-value^a^
		Inadequate (0%–53%)	Moderate (54%–66%)	Adequate (67%–100%)	
		*n* (%)	*n* (%)	*n* (%)	
**Gender**					
Male	114	30 (26.3)	30 (26.3)	54 (47.4)	0.233
Female	359	74 (20.6)	83 (23.1)	202 (56.3)	
**Ethnicity**					
Malay	399	92 (23.1)	100 (25.1)	207 (51.9)	0.185
Chinese	45	7 (15.6)	8 (17.8)	30 (66.7)	
Other	15	3 (20.0)	1 (6.7)	11 (73.3)	
**Age (in years)**					
18–29	305	58 (19.0)	68 (22.3)	179 (58.7)	0.228
30–39	66	16 (24.2)	17 (25.8)	33 (50.0)	
40–49	38	11 (28.9)	9 (23.7)	18 (47.4)	
≥50	45	14 (31.1)	13 (28.9)	18 (40.0)	
**Education**					
High school	79	28 (35.4)	26 (32.9)	25 (31.6)	<0.001
College	71	14 (19.7)	17 (23.9)	40 (56.3)	
Bachelor’s degree	188	33 (17.6)	34 (18.1)	121 (64.4)	
Postgraduate (master’s)	50	5 (10.0)	10 (20.0)	35 (70.0)	
Others	84	23 (27.4)	26 (31.0)	35 (41.7)	
**Medical history of genetic disease**					
No	463	103 (22.2)	114 (24.6)	246 (53.1)	0.038
Yes	11	1 (9.1)	0 (0.0)	10 (90.9)	
**Family history of genetic disease**					
No	449	102 (22.7)	108 (24.1)	239 (53.2)	0.193
Yes	25	2 (8.0)	6 (24.0)	17 (68.0)	

^a^ Chi-squared or Fisher’s exact test.

### 3.3 Perceived genetic knowledge

In total, 60.9% of the participants reported to demonstrate some perceived knowledge (either little or sufficient) on genetics and genetic testing (Table 5). However, significantly less (*p* = 0.025) participants knew about the social consequences (54.0%) compared to the medical possibilities (65.5%) (Table 5). Out of all the medical possibilities of genetic testing, 80.1% participants were most familiar with the potential of testing in the early detection of specific diseases. On the social side, 61.3% of participants exhibited some knowledge on their rights to decline genetic testing. However, 50.4% of the participants showed no information on the rights of third parties over the results of a genetic test. The percentage response for each statement in Part 3 was compared to an American study (Haga et al., 2013) (Table 5). Our study reported a lower percentage mean response for “little” knowledge for both medical possibilities and social consequences (*p* = 0.023, Table 5). No significant difference was noted in overall mean scores with respect to gender, ethnicity, age group, education level, and personal medical and family history of genetic disease(s) (Table 6).

**TABLE 5:** Participants’ percentage response pertaining to perceived knowledge of genetics.

Statement	Nothing		Little		Sufficient	
*‘How much do you know about…’*	*Current study*	*Haga et al., 2013* *^a^*	*Current study*	*Haga et al., 2013* *^a^*	*Current study*	*Haga et al., 2013* *^a^*
Medical possibilities						
1. The possibility of early detection of certain disorders by genetic testing	19.9	14	64.3	68	15.8	18
2. The significance of genetic testing for your relatives	31.6	21	48.6	64	19.8	15
3. The significance of genetic testing for your offspring	28.1	18	44.4	63	27.4	19
4. The possibility of genetic knowledge to prevent or treat a disorder	22.9	15	52.7	67	24.3	19
5. The possibilities and risks of gene therapy	36.9	38	45.9	51	17.3	11
6. Breast cancer genetic testing	30.0	-	45.6	-	24.3	-
7. The ability of breast cancer genetic tests to detect mutations in *BRCA 1* and *BRCA2* genes that normally function to suppress tumor growth	43.2	-	38.9	-	17.9	-
8. Colorectal cancer genetic testing	49.1	-	37.3	-	13.7	-
9. The ability of colorectal cancer genetic tests to detect the familial type of colorectal cancer	49.3	-	39.6	-	11.1	-
Subsection mean response	34.6	21	46.4 ^bc^	63 ^c^	19.1 ^b^	16
Social consequences						
10. Your rights to refuse genetic testing	38.7	19	42.7	49	18.6	32
11. The consequences of genetic testing for your daily life	45.3	29	38.9	54	15.8	17
12. The consequences of genetic testing for your work	50.1	45	36.2	49	13.7	15
13. The consequences of genetic testing for taking out insurance	49.5	34	38.3	48	12.2	17
14. Your own possibilities to apply for a genetic test	41.6	39	44.0	46	14.4	15
15. The rights of third parties to inquire about the results of a genetic test	50.4	50	36.5	38	13.1	12
Subsection mean response	45.9	36	39.4 ^bc^	46 ^c^	14.6 ^b^	18
Overall mean response	39.1	28	43.6	54	17.3	17

^a^ Study population consisted of 300 individuals enrolled from Durham, NC, with no personal history of type 2 *diabetes mellitus* (T2DM), and had not had a genetic test for T2DM.

^b^ Differences between knowing a little and sufficient knowledge on medical possibilities and social consequences were significant at *p*=0.025.

^c^ Differences between the two populations’ response in having a little knowledge on medical possibilities and social consequences were significant at *p*=0.023.

**TABLE 6:** *p*-value for the respective variables pertaining to questions on perceived knowledge of genetics and attitude toward genetic testing.

Variable	Perceived knowledge			Attitude		
	*Overall score*			*Overall score*		
	*n*	Mean (SD)	*p*-value	*n*	Mean (SD)	*p*-value
**Gender**						
Male	101	25.9 (7.06)	0.332^a^	98	46.2 (4.76)	0.687^a^
Female	322	26.8 (7.58)		313	46.0 (4.64)	
**Ethnicity**						
Malay	357	26 (11)	0.432^b^	344	46 (6)	0.153^b^
Chinese	39	29 (12)		39	47 (5)	
Other	13	(27 (17)		14	45 (4)	
**Age (in years)**						
18–29	277	26.7 (7.46)	0.112^c^	268	46.2 (4.50)	0.001^c, d^
30–39	60	25.8 (7.86)		59	46.0 (3.99)	
40–49	32	29.3 (6.85)		32	47.8 (5.55)	
≥50	36	25.3 (7.53)		34	43.2 (5.61)	
**Education**						
High school	75	26.4 (7.64)	0.333^c^	71	46.4 (5.64)	0.117^c^
College	65	26.9 (7.14)		63	44.5 (4.57)	
Bachelor’s degree	160	26.4 (7.43)		157	46.2 (4.40)	
Postgraduate (master’s)	44	24.8 (8.39)		42	46.3 (4.42)	
Others	78	27.7 (7.09		77	46.1 (4.38)	
**Medical history of genetic disease**						
No	415	26 (12)	0.939^e^	402	46 (6)	0.722^e^
Yes	9	26 (10)		10	46 (7)	
**Family history of genetic disease**						
No	400	26 (12)	0.791^e^	387	46 (6)	0.726^e^
Yes	24	25 (10)		25	46 (5)	

SD, standard deviation.

^a^ Independent *t*-test.

^b^ Kruskal–Wallis test, values are presented as median (interquartile range).

^c^ One-way ANOVA test.

^d^ Age group 18–29 or 40–49 and ≥50 years old pairs of mean scores are significantly different by the *post hoc* test (Scheffe’s procedure).

^e^ Mann–Whitney test, values are presented as median (interquartile range).

Analysis on each statement in Part 3 revealed majority of the participants (52.7%, Table 5) have little knowledge on the possibility of genetic knowledge to prevent or treat a disorder; participants with a family medical history of genetic disease know more than participants without a family history (*p* = 0.042). However, 49.5% (Table 5) of the participants do not know the consequences of genetic testing for taking out insurance; participants aged 40–49 years old reported more knowledge than participants aged 18–29 years old (*p* = 0.026).

### 3.4 Attitude toward genetic testing

Majority of the participants agreed more with statements associated with favorable attitudes than those with reserved attitudes (Table 7). Notably, participants agreed most that the development of DNA research is a positive progress in the medical field (82.8%, Table 7). In total, 80.3% of the participants believed that genetic tests could alter one’s future. When asked about informing their children about their genetic testing results for a specific disease, 63.8% of the participants would do so, while 69.4% would inform their siblings. Overall, the participants showed relatively good attitudes toward genetic testing, which is influenced by their age (*p* = 0.001, Table 6), where younger participants are open to the idea of genetic testing compared to older participants.

**TABLE 7:** Participants’ percentage response pertaining to attitudes toward genetic testing.

Statement	*n* (%)				
	Totally disagree	Disagree	Neutral	Agree	Totally agree
Favorable attitudes					
1. I think the development of DNA research is hopeful for the treatment of diseases	2 (0.5)	5 (1.2)	91 (22.1)	221 (53.6)	93 (22.6)
2. I think that the development of DNA research is a positive medical progress	1 (0.2)	4 (1.0)	66 (16.0)	234 (56.8)	107 (26.0)
3. I approve of using genetic-testing for early detection of diseases	2 (0.5)	12 (2.9)	62 (15.0)	234 (56.8)	102 (24.8)
4. I would inform my children about the results of a genetic test for a specific disease	3 (0.7)	15 (3.6)	131 (31.8)	214 (51.9)	49 (11.9)
5. I want to know whether my disease is hereditary	-	11 (2.7)	80 (19.4)	216 (52.4)	105 (25.5)
6. I would inform my siblings about the results of a genetic test for a specific disease	4 (1.0)	12 (2.9)	109 (26.7)	232 (56.7)	52 (12.7)
Reserved attitudes					
7. I worry about the consequences of genetic testing for being able to take out insurance	4 (1.0)	28 (6.8)	214 (51.9)	126 (30.6)	40 (9.7)
8. The possibility of a genetic test will change one’s future	1 (0.2)	5 (1.2)	75 (18.2)	233 (56.6)	98 (23.8)
9. As long as a disease cannot be treated, I do not want a genetic test	24 (5.8)	112 (27.2)	172 (41.7)	82 (19.9)	22 (5.3)
10. If I had a genetic test conducted, my family need not know about the result	33 (8.0)	120 (29.2)	125 (30.4)	96 (23.4)	37 (9.0)
11. I do not want a genetic test to tell me that I am at risk for a certain disease	45 (10.9)	154 (37.4)	124 (30.1)	68 (16.5)	21 (5.1)
12. I worry about the consequences of genetic testing for the chances of finding a job	12 (2.9)	62 (15.0)	126 (30.6)	155 (37.6)	57 (13.8)
13. The idea of a genetic test frightens me	32 (7.8)	99 (24.0)	141 (34.2)	108 (26.2)	32 (7.8)

Notwithstanding the former finding, analysis on each statement in Part 4 revealed that the adolescents (18–29 years old) displayed higher concerns (*p* = 0.011) on the impact of genetic testing on their employment prospects than the older population (50 years old or above) and were more frightened by the idea of a genetic test (*p* = 0.041). It was also observed that the Chinese participants were more likely to want to know the hereditary nature of their disease compared to the Malay participants (*p* = 0.05). Individuals with a personal medical history of genetic disease also showed significantly higher curiosity than individuals with no personal history (*p* = 0.008). Participants with a master’s degree significantly agreed on the promising progress of DNA research than participants with other educational backgrounds (*p* = 0.006). Significant differences were also noted between participants with high school education, who were more reluctant to take a genetic test for an untreatable disease, and those with college education (*p* = 0.007) or a bachelor’s degree (*p* = 0.024) education. On that note, less proportion of participants with a family history of genetic disease, than those without, agreed to the statement that they would not want a genetic test for an untreatable disease (*p* = 0.037).

## 4 Discussion

Research in the area of genetics and genetic testing possesses vast potential in the field of personalized medicine. Genetic testing could provide early detection and prevention of diseases (Grosse and Khoury, 2006). However, utilization and optimization of such testing in clinical care settings only arrive upon certain factors including acceptance and accurate understanding (Kinney et al., 2010). Routine genetic testing is not conducted in Brunei Darussalam yet, and little is known locally about the level of knowledge, direction of attitudes, and their determining factors, surrounding genetic literacy and testing in this country. This study aimed to investigate the population’s actual and perceived knowledge on genetics and genetic testing, as well as attitudes, and the possible importance of these findings in relation to Brunei. The results from this study could contribute to the general picture of current knowledge on genetics and more importantly, facilitate national decisions on the use of genetic research and testing for its role in health and disease.

Assessment of actual knowledge of genetics on topics such as genes, chromosomes, and disease revealed an overall mean score of 69.4%. This score is lower than that of the study conducted in America that showed an overall average score of 83.6% (Haga et al., 2013), of which the questionnaire was adapted from. The observed disparity could be explained by the differences in socio-economy, culture, education curriculum, and health services provided including availability of genetic testing and counseling (Amin et al., 2012; Haga et al., 2013). Necessary genetic testing for patients in Brunei is performed overseas such as in Singapore. This part of the healthcare system could suggest a lack of national exposure to the realm of genetics and the remoteness of such a subject to the average public. In terms of education, the concept of genetics is introduced in year 11 (high school) in Brunei and only expanded if specific subjects, like Biology, are majored during further studies; otherwise, knowledge about genetics is dependent on the individual’s own interest. This is a contrast to the increased penetration of genetics into the American culture causing enhanced public familiarity (Bates, 2005). When compared to our neighboring countries, such as Malaysia, they also showed adequate knowledge of genetic testing (Chin and Tham, 2020), increasing the public familiarity of testing despite having similar culture. However, a German report states that public familiarity does not guarantee understanding. In addition, substantial information could also generate confusion and misconception (Berth et al., 2002).

Our study revealed that the younger participants demonstrated higher actual knowledge than the older participants, which is consistent with other published studies (Jallinoja and Aro, 1999; Calsbeek et al., 2007; Haga et al., 2013; Henneman et al., 2013). In addition to the knowledge from what they learn at school, the younger participants are also potentially exposed to information gathered from the internet, including those about genetic testing and its benefits (Covolo et al., 2015).Our results also established that a higher educational background of participants was associated with a higher level of genetic knowledge, which is also found in the previous report (Calsbeek et al., 2007; Chapman et al., 2019).Our study did not ask for the participants’ field of learning when they were in the higher education (university level). It would be interesting to see whether the higher level of genetic literacy findings in the public was related to their field of study, a finding that was found in a study conducted focusing on medical students in Indonesia (Rujito et al., 2020; Swandayani et al., 2021). Increased scientific research and, in the context of Brunei, the reformations in education and school systems (namely, bilingual education and SPN-21) through the years are likely to have played a part in the differences of knowledge exposed to the distinct generations. Moreover, the study reported that elderly people are also less likely to be influenced by the media (Calsbeek et al., 2007). It was observed that our study population exhibited more knowledge on disease-related concepts compared to scientific facts, which is consistent with the previous studies (Jallinoja and Aro, 1999; Calsbeek et al., 2007; Haga et al., 2013). A possible explanation for this finding is that people tend to retain longer memory on information that has more obvious effects on themselves than facts that seem irrelevant.

The level of perceived knowledge of the participants in this study is significantly lower than the participants in the American study (Haga et al., 2013) but higher than that of the Dutch study (Morren et al., 2007). It was hypothesized that one of the reasons for low perceived knowledge is that people are becoming more aware that their individual knowledge is only a minute fraction of what is known (Calsbeek et al., 2007). Moreover, the relationship between subjective evaluations of own knowledge and objective knowledge is suggested to be unspecified (Morren et al., 2007). In spite of this, motivation to find more genetic information would be inspired by perceived knowledge.

Knowledge assumes a vital position in the translation of genetic testing from the research bench to the healthcare venue. Higher degrees of knowledge ensure informed decision making (Haga et al., 2013). Inadequate information about genetics and genetic testing may be causing people to avoid taking a genetic test when necessary, leading to poorer health, reduced quality of life, and increased medical costs when an easily preventable disease requires later treatment (Morren et al., 2007). Education on genetics and genetic testing could be carried out by clinicians, for example, general practitioners (GP), which is the preferred source of genetic information (Morren et al., 2007). However, it was reported that GPs and even clinical geneticists expressed a lack of confidence in their genetic expertise and, consequently, their ability to provide such information to patients (Kampourakis, 2017). Preparation of genetic testing in Brunei, thus, not only takes into account public readiness but also proper training or qualification of healthcare professionals should also be prioritized, which include the availability of a qualified genetic counselor (Matusin, 2020). Such training for healthcare providers must see meticulous tailoring in the knowledge of and skills to perform the genetic test itself, and understanding of the ethical issues on the access of the information to be provided for the general population.

Participants with a family medical history of genetic disease displayed a higher interest in genetic testing, even for an untreatable disease, than participants without a family history. Research has found conflicting associations between a familial history and interest in breast cancer genetic testing, reporting positive (Kash and Dabney, 2001), negative (Andrykowski et al., 1997), and no associations (Donovan and Tucker, 2000) between the two. However, high interest in genetic testing is not synonymous with true demand for testing (Bruno et al., 2004) as it could stem from interest in modern laboratory procedures, inappropriate knowledge of genetic testing, or curiosity-driven behavior (Bottorff et al., 2002).

This study was limited by the missing data across all parts of the questionnaire. For data collection conducted in public areas, allowing face-to-face interaction with participants, it was observed that not all participants displayed interest on the topic of genetics despite attempting the questionnaire. This lack of motivation makes it difficult to assess the true knowledge level of participants. In addition to the lack of interest, missing data could be attributed to inconvenient timing or difficulty of the questionnaire. Another limitation is the answer choices for the actual knowledge of genetics. A correct response for this section could be merely coincidental or guess work. Further use of this survey instrument could include an “I do not know” option revealing the true lack of knowledge or lack of confidence in one’s knowledge. The questionnaires for this study were also made available through social media, which might help account for the population who do not go to the visited frequented areas, and added to the strength of this study, which is the large sample size. Participants from the Chinese and other ethnic groups only make up about 10% of the sample size and males around 20%, which is not representative of the Bruneian population. Further research could ensure appropriate distribution of the study across ethnicity and gender.

In conclusion, the full potential of genetics and genetic testing in health and disease is currently not channeled to the world population. This bottleneck phenomenon could be due to the variation of knowledge and attitudes held by people surrounding testing, owing to the many influences such as media, service provision agencies, and scientific publication. Thus, assessment of public knowledge and attitudes of the consumers of genetic testing is a great stepping stone toward tailoring the nation’s lens so as to reap the benefits of this genetics era.

## Data Availability

The original contributions presented in the study are included in the article/Supplementary Material; further inquiries can be directed to the corresponding author.
